# Chitosan/Gelatin/Silver Nanoparticles Composites Films for Biodegradable Food Packaging Applications

**DOI:** 10.3390/polym13111680

**Published:** 2021-05-21

**Authors:** Sreelekha Ediyilyam, Bini George, Sarojini Sharath Shankar, Thomas Thuruthiyil Dennis, Stanisław Wacławek, Miroslav Černík, Vinod V. T. Padil

**Affiliations:** 1 Department of Chemistry, School of Physical Sciences, Central University of Kerala, Kasaragod 671316, Kerala, India; sreelekha.e469@gmail.com; 2 Department of Biochemistry and Molecular Biology, School of Biological Sciences, Central University of Kerala, Kasaragod 671316, Kerala, India; 3 Department of Medicine, Thomas Jefferson University, Jefferson Alumni Hall, 1020 Locust Street, Philadelphia, PA 19107, USA; 4 Department of Plant Science, School of Biological Sciences, Central University of Kerala, Kasaragod 671316, Kerala, India; den_thuruthiyil@cukerala.ac.in; 5 Department of Nanomaterials in Natural Sciences, Institute for Nanomaterials, Advanced Technologies and Innovation (CXI), Technical University of Liberec (TUL), Studentská 1402/2, 461 17 Liberec 1, Czech Republic; stanislaw.waclawek@tul.cz

**Keywords:** chitosan, gelatin, silver nanoparticles, bio-nanocomposites, antimicrobial, food packaging

## Abstract

The food packaging industry explores economically viable, environmentally benign, and non-toxic packaging materials. Biopolymers, including chitosan (CH) and gelatin (GE), are considered a leading replacement for plastic packaging materials, with preferred packaging functionality and biodegradability. CH, GE, and different proportions of silver nanoparticles (AgNPs) are used to prepare novel packaging materials using a simple solution casting method. The functional and morphological characterization of the prepared films was carried out by using Fourier transform infrared spectroscopy (FTIR), UV–Visible spectroscopy, and scanning electron microscopy (SEM). The mechanical strength, solubility, water vapor transmission rate, swelling behavior, moisture retention capability, and biodegradability of composite films were evaluated. The addition of AgNPs to the polymer blend matrix improves the physicochemical and biological functioning of the matrix. Due to the cross-linking motion of AgNPs, it is found that the swelling degree, moisture retention capability, and water vapor transmission rate slightly decrease. The tensile strength of pure CH–GE films was 24.4 ± 0.03, and it increased to 25.8 ± 0.05 MPa upon the addition of 0.0075% of AgNPs. The real-time application of the films was tested by evaluating the shelf-life existence of carrot pieces covered with the composite films. The composite film containing AgNPs becomes effective in lowering bacterial contamination while comparing the plastic polyethylene films. In principle, the synthesized composite films possessed all the ideal characteristics of packaging material and were considered biodegradable and biocompatible food packaging material and an alternate option for petroleum-based plastics.

## 1. Introduction

Among the numerous current-day nanotechnologies, polymer nanocomposite has been given more attention in the food packaging industry. It possesses improved packaging properties, including moisture permeability, barrier properties, oxygen permeability, solvent resistance, thermal stability, anti-microbial, and mechanical sturdiness [1]. Packaging films using biopolymer have been viewed as substitutes for conventionally used petroleum-derived plastic packaging material. Disposal of conventional petroleum-based polymeric materials may create significant issues in the environment and health. It is necessary to have an alternate packaging material. Polysaccharide-based biopolymers have considerable attention in the packaging industry since they are biodegradable, biocompatible, cheap, non-toxic, renewable, and edible [2]. They degrade under appropriate conditions of moisture, temperature, and oxygen availability and limit toxic residue production [3], which may contaminate the environment.

Biopolymer gelatin (GE) derived from collagen has properties like gel formation, texturizing, thickening, etc. The emulsifying property of GE expands its use in the food industry, and it is used in a variety of food stuff [4]. The opulent film-forming ability [5] of GE has been considerably used to preserve the food from the external environment, such as light, air, and microorganisms. Hence, it is a magnificent food packaging material and helps lengthen the shelf life of food materials. GE contains a chain of aromatic amino acids [4] to promptly absorb UV light and obstruct light into the food materials. However, its low thermal strength, elasticity, and mechanical properties limit the possible applications in different filed [6]. To overcome these limitations combination of various additives, including synthetic and biopolymer like chitosan (CH) [7], starch [8], or pectin [9], can be used. CH is a cationic biopolymer and natural biomass origin intrinsically biocompatible, biodegradable, and antimicrobial [10]. It is extensively explored in various fields such as powder, films, membranes, and sheets [11]. Inherently antimicrobial CH [12] hinders the entrance and formation of microorganisms into the food materials; hence, it is preferably used for food packaging applications. Besides, it has better mechanical and gas barrier properties [13]. The composite films of CH and GE display advanced mechanical, thermal, barrier, and physicochemical properties compared to the films with mono-biopolymer, either CH or GE. Nowadays, research focuses on developing active polymer packaging films with antioxidant and antimicrobial additives to increase the biological features of food stuff [14]. Peppermint and citronella essential oils are used to increase the antimicrobial properties of GE films [15]. To impart antioxidant and antimicrobial properties to the films mainly essential oil and extracts are used as additives [13].

Nanofillers incorporate the polymer matrix to improve physicochemical properties such as barrier, hydrophobicity, and conductive properties. Plant-mediated synthesis of silver nanoparticles (AgNPs) is a simple, cost-effective, and eco-friendly method [16]. It has significant advantages over the other chemical synthesis of nanoparticles, particularly to many points like the ease of preparation, rate of formation, health, environmental care, capital investment, etc. AgNPs are effective against fungi [17] and a wide range of Gram-negative and Gram-positive bacteria [18]. Due to the low toxicity of AgNPs [18] with antioxidant [16] and antibacterial properties, it has been used in diverse applications in different sectors. The incorporation of nanofillers to the polymer blend matrix alters mechanical and barrier properties and accelerates other biological features such as antimicrobial, biosensing, and oxygen scavenging properties [19].

*Mussaendafrondosa L.* [*M. frondosa*] is a medicinal plant belonging to the family Rubiaceae. Traditionally it has been used to treat ulcers, jaundice, wounds, bronchitis, cough, white leprosy, skin infections, eye troubles, and tuberculosis. Plants contain many biomolecules such as phenols, flavonoids, alkaloids, steroids, glycosides, tannins, etc. [20]. Carbohydrates, tannins, alkaloids, flavonoids, and polyphenols are the primary chemical moiety present in the aqueous extract of *M. frondosa* [21]. On account of its precious medicinal properties, this plant is mainly used for biomedical purposes.

The present study mainly focused on preparing biogenic, non-toxic AgNPs incorporated polymer blend matrix for active food packaging applications. Antioxidant and antibacterial AgNPs were synthesized using lyophilized aqueous extract of *M. frondosa*. The nanocomposite films were prepared by incorporating AgNPs to the CH–GE polymer matrix. Mechanical, antibacterial, biodegradability, transparency, and physiochemical properties of synthesized nanocomposite films were assessed and compared. The incorporation of AgNPs increases the biological functioning of films, which is evaluated through extent of the shelf life of carrot pieces wrapped in them.

## 2. Materials and Methods

### 2.1. Materials

Silver nitrate (AgNO_3_) was purchased from Merck Chemicals, Mumbai, India. CH with deacetylation of ≥90% and GE were purchased from Hi-Media Laboratories Pvt. Ltd. (Mumbai, India). Polyethylene glycol (PEG, M_w_-500) and acetic acid were purchased from Loba Chemie Pvt. Ltd., Mumbai, India. Millipore water was used to prepare all the aqueous solutions. The leaves of *M. frondosa* were collected from the forest area of Mulleriya, located in Kasaragod district, Kerala, India.

### 2.2. Methods

#### 2.2.1. Preparation of Plant Extract

Freshly collected *M. frondosa* leaves were shade dried and powdered using mortar and pestle. Then, 10 g of the powdered sample was mixed in 300 mL of Millipore water, and the mixture was shaken for 6 h in an orbital shaker (Orbital shaker, Sub-Zero, Chennai, India). Then, the extract was double-filtered using a muslin cloth and centrifuged at 8000× *g* rpm for 15 min (centrifuge machine, Eppendorf, Chennai, India). The supernatant solution was collected and lyophilized. The lyophilized sample was further used for the preparation of AgNPs.

#### 2.2.2. Preparation of AgNPs

AgNPS were prepared by adding 1 mL of *M. frondosa* leaf extract (10 mg/mL) to 10 mL of AgNO_3_ solution (10 mM). The resulting solution was held at 80 °C in a magnetic stirrer for 1 h. The color of the solution changed from light yellow to reddish-brown, indicating the formation of AgNPs. This colloidal solution was lyophilized and used in subsequent research.

#### 2.2.3. Preparation of Composite Films

The solution casting method was used to prepare the CH–GE–AgNPs composite films described by Santosh Kumar et al., which was slightly modified by incorporating lyophilized AgNPs and changing the preparation technique [22]. 2 g of CH was dissolved in 100 mL of 1% acetic acid and stirred for 12 h to produce a homogeneous CH solution. The resultant solution was further filtered using cheesecloth to remove the undissolved particle. 2% of gelatin was prepared by dissolving 2 g of GE in 100 mL of water. To get a fine morphology, we blended the films with the composition of CH: GE (90:10) as reported by Ahmed et al. [23]. This mixture was placed on the orbital shaker with continuous shaking for 1 h (Orbital shaker, Sub-Zero, Chennai, India). After that, 0.5 g of polyethylene glycol was added to the homogeneous mixture, which was stirred for a further 3 h. The resulting homogenous mixture was transferred to a Petri dish and placed at room temperature for evaporation. The films were then peeled from the Petri dishes and stored in a desiccator for further use. To obtain CH–GE–AgNPs films, AgNPs with varying concentration were added to the CH–GE mixture, and the above procedure was repeated (Table 1).

### 2.3. Characterization of Nanoparticles and Composite Films

#### 2.3.1. UV–Visible Spectrophotometer

UV–Visible spectroscopy was used to conduct preliminary characterization of the AgNPs. Perkin Elmer UV–Visible Spectrometer Lambda-35 (Perkin-Elmer SCIEX, Waltham, MA, USA) was used to record UV–Visible spectra scanned from 200 to 800 nm.

#### 2.3.2. Fourier Transform Infrared (FTIR) Spectroscopy

The FTIR spectra were acquired using a Perkin–Elmer Spectrum-Two instrument (Perkin–Elmer SCIEX, Waltham, MA, USA), scanning the 400 to 4000 cm^−1^ range.

#### 2.3.3. Morphological Analysis by TEM

A high-resolution transmission microscope, the Jeol Model JM 2100 (JEOL, Tokyo, Japan) was used for the TEM experiments, which had a resolution point of 0.23 nm and a lattice of 0.14 nm and was worked at an accelerating voltage of 200 kV.

#### 2.3.4. Scanning Electron Microscopy (SEM)

The JEOL JSM 6390 (JEOL, Tokyo, Japan) was used to conduct surface morphology observations. On aluminum stubs, samples were taped together with double-sided carbon tape. The images were analyzed at a 10 kV accelerating voltage after all specimens were sputtered with a thin layer of gold in an auto fine coater JEOL JFC 1600 (JEOL, Tokyo, Japan).

#### 2.3.5. Mechanical Strength (UTM)

The films’ tensile strength and elongation at break were determined using a Universal Testing Machine (SHIMADZU AG-X plus, Shimadzu, Kyoto, Japan) and followed ASTM standard method D882. For the study, a test composite with almost equal thickness was used. The thickness of the film samples was measured with a digital micrometer at six different random locations, and the mean values were reported as the average.

### 2.4. Evaluation of the Antioxidant Activity of AgNPs by DPPH Radical Scavenging Method

Ethanol was used for the preparation of 0.01 mM DPPH solution. A volume of 1 mL of DPPH solution was mixed with 5 mL of green synthesized AgNPs solution at various concentrations (5 µg/mL to 200 µg/mL). The absorbance was taken at 517 nm using a UV–Visible spectrometer after the mixture was continuously shaken and allowed to stand at room temperature for around 15 min. The relatively low absorbance of the reaction mixture indicates the higher DPPH scavenging activity. The percent DPPH scavenging effect was calculated using the equation below.

(1) $$\%\;\mathrm{of}\;\mathrm{inhibition}=\frac{\mathrm{Absorption}\;\mathrm{of}\;\mathrm{control}-\mathrm{Absorption}\mathrm{of}\;\mathrm{test}}{\mathrm{Absorption}\;\mathrm{of}\;\mathrm{control}}\times 100$$

### 2.5. Antibacterial and Antifungal Activity of AgNPs

The antibacterial behavior of AgNPs was examined using the well diffusion method. Muller–Hinton Agar Medium was used for the bacterial culture of *Staphylococcus aureus (S. aureus*) (ATCC 25923), *Escherichia coli (E. coli)* (ATCC 25922), *Streptococcus mutans* (*S. mutans*) (ATCC 25175), and *Pseudomonas aeroginosa* (*P. aeroginosa*) (ATCC 27853). In a bacterial culture, plate wells with almost equal diameter were made, and 10 μL of different concentrations of AgNPs was added to each well and incubated overnight at 37 °C for 24 h [24]. The zone of inhibition was determined after overnight incubation of the culture plate and compared with the standard Streptomycin.

*Candida albicans* (*C.albicans*) (ATCC 10231) were cultured in Potato Dextrose Agar plates. Wells of almost equal diameter were carved, and 10 μL samples of varying concentrations of AgNPs were applied to the various wells [25]. After overnight incubation at room temperature, the zone of inhibition was assessed and compared to that of typical antimycotic drugs (Clotrimazole) (NCCLS, 1993).

### 2.6. Film Thickness

Using a digital micrometer screw gauge (MSGD-025-SN, Ajanta Export Industry, Haryana, India) with a precision of 0.01 mm, the thickness of the film samples was determined at six different random points, and the mean values were recorded as the average thickness.

### 2.7. Apparent Density

The apparent density of the films was measured as described by Lozano-Navarro et al. [26]. The films with almost equal thickness were cut in a circular shape and weighed. The apparent density (ρ) of films was calculated using the following equation:

(2) $$\rho =\frac{W}{\left(\pi \times {\left(\frac{D}{2}\right)}^{2}\times H\right)}$$

W—Weight of the sample (g)

D—Diameter of the sample (cm)

H—Height of the sample (cm)

### 2.8. Solubility

The films (thickness of 2 cm × 2 cm) were cut and weighted (W_a_) to determine their solubility and then placed in a beaker with 50 mL of distilled water. The beaker was wrapped and kept at 25 °C for 24 h on a shaking incubator. The films were dried at 40 °C in the oven until a constant weight (W_x_) of films was obtained. It was calculated as follows [27]:

(3) $$\mathrm{Solubility}\;\left(\%\right)=\frac{\left(W_{a}-W_{x}\right)}{W_{a}}\times 100$$

### 2.9. Swelling Degree (%W)

The discrepancy between the weights of the dry film and the weight of the films after immersion in distilled water for 24 h at 37 °C and subsequent elimination of surface water by blotting with tissue paper is used to calculate the swelling degree (%) of the films [28]. It was calculated as follows:

(4) $$\mathrm{EDS}\;\left(\%\right)=\frac{W_{s}-W_{d}}{W_{d}}\times 100$$

W_d_—Weight of the dried sample(g)

W_s_—Weight of the sample after immersion in water(g)

### 2.10. Water Vapour Transmission Rate [WVT]

To test the WVT of films, bottles with a mouth diameter of 1.5 cm were used and loaded with 10 mL of deionized water. Polymer films were used to cover the bottle’s mouth, which was then air tightened with Teflon tape. The bottle was weighed and kept in the oven for 24 h at 40 °C. The bottle was removed from the oven after 24 h and weighed again [29]. WVT was calculated as follows in (g/m^2^h).

(5) $$\mathrm{WVT}=\frac{W_{i}-W_{t}}{A\times t}$$

W_i_—Initial Weight (g)

W_t_—Final Weight (g)

A—Mouth area of the bottle (m^2^)

t—Time (h)

### 2.11. Test of Biodegradability

For soil burial decay, the test samples were buried for two weeks in a pot of soil (40–45% of inorganic matter, 5% of organic matter, and 25% of air and water) at a depth of 10 cm. The pot was placed in the laboratory and to keep the soil moist the water was sprayed regularly. The pot’s excess water was drained through a hole in the bottom. The samples’ decay was assessed at regular intervals.

The weight loss was calculated using the equation below.

(6) $$\mathrm{Weight}\;\mathrm{loss}\;\left(\%\right)=\frac{W_{f}-W_{d}}{W_{f}}\times 100$$

W_f_—Final weight of the films (g)

W_d_—Dry weight of the films (g)

### 2.12. Moisture Retention Capability

The moisture-retaining capability of the films was measured by cutting and weighing films of nearly equal thickness. These films were kept in the oven for 6 h. The films were removed from the oven after 6 h and weighed again [28]. The formula was used to determine the moisture-retaining ability was given below.

(7) $$\mathrm{Moisture}\;\mathrm{retention}\;\mathrm{capability}\;\left(\%\right)=\frac{W_{t}}{W_{i}}\times 100$$

W_t_—Final Weight of the test films (g)

W_i_—Initial Weight of the test films (g)

### 2.13. The Potential Ability of the Synthesized Film towards Food Packaging Applications

Food packaging applications of fabricated films were analyzed using the spread plate method described in Saral Sarojini et al. [30]. The cut piece of carrot with the same size, shape, and color was used for food packaging testing of composite films. Pure CG and CG4 films were freshly fabricated and taped around each cut piece of carrot. Plastic (polyethylene) film was used as a control to cover carrot pieces and kept undisturbed. The carrot samples were examined using the serial dilution and spread plate procedure on the tenth day of storage. Then, 1 g of carrot in 10 mL of sterile distilled water was used to make 10^−1^ microbial suspension. Serial dilutions of the microbial suspension were done. A volume of 0.1 mL of suspensions from the dilutions were spread plated over the surface of sterile nutrient agar medium and incubated for 24 h at room temperature. The isolated colonies were counted by the colony-forming unit (CFU) method after 24 h of incubation [31].

## 3. Results

### 3.1. Surface Functionality Analysis by UV–Visible and Fourier-Transform Infrared Spectroscopy

**Figure 1:**
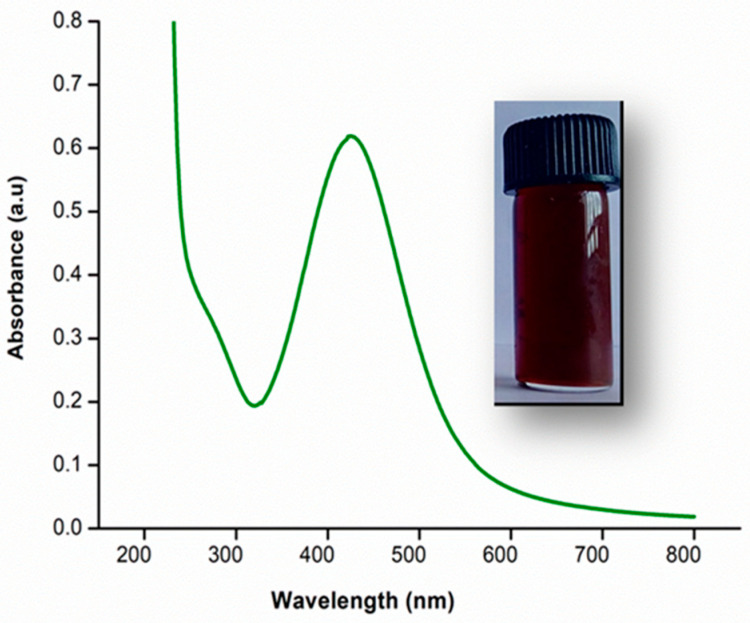
UV–Visible spectral analysis of green synthesized AgNPs.

UV–Visible spectroscopy was used to verify the nanoparticles’ formation and stability. It was also used to determine the reaction progress and completion. Surface plasma resonance (SPR) of AgNPs ranges between 390–470 nm [32]. The synthesized AgNPs from the leaf extract of *M. frondosa* showed an absorption peak at 426 nm. It primarily confirms the formation of AgNPs. It is demonstrated in Figure 1.The series of changes during AgNPS synthesis was in Figure S1 (Supplementary Materials).

**Figure 2:**
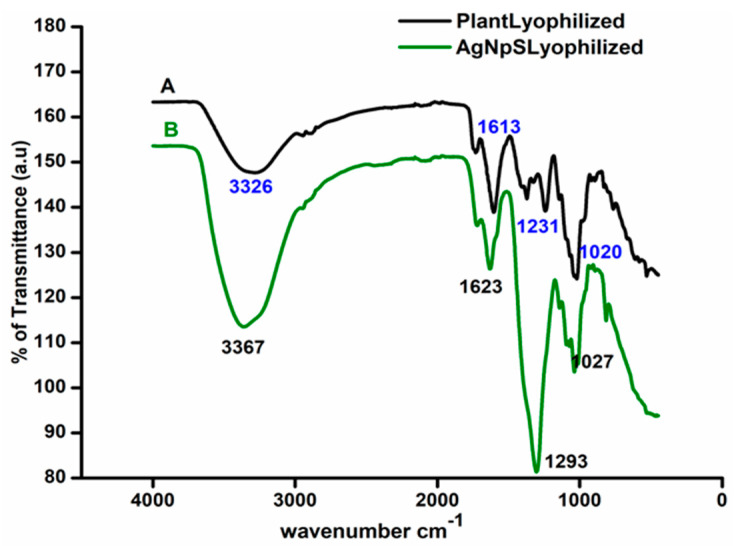
FTIR spectra of (**A**) Lyophilized aqueous *M.frondosa* leaf extract (**B**) Lyophilized AgNPs.

FTIR studies confirmed the surface functionality of the synthesized nanoparticles. Figure 2A,B display FTIR spectra of *M. frondosa* leaf extract and green synthesized AgNPs with nearly identical IR bands. Leaf extract showed vibration bands at 3326, 1612, 1231, and 1020 cm^−1^ corresponding to O–H stretching of an aromatic alcohol, amide I band, C–O stretching of alcohol, and C–N stretching vibration of the amine, respectively. In the case of AgNPs, the absorbance peak shifted from 3326 to 3367 cm^−1^ and from 1231 to 1296 cm^−1^ with increased band intensity, suggesting silver ion binding to the extract’s hydroxyl and carboxylate groups [33]. A change in the wavenumbers corresponding to amide I (1612–1623 cm^−1^) linkages was also visible in the FTIR spectra.

### 3.2. TEM Analysis

**Figure 3:**
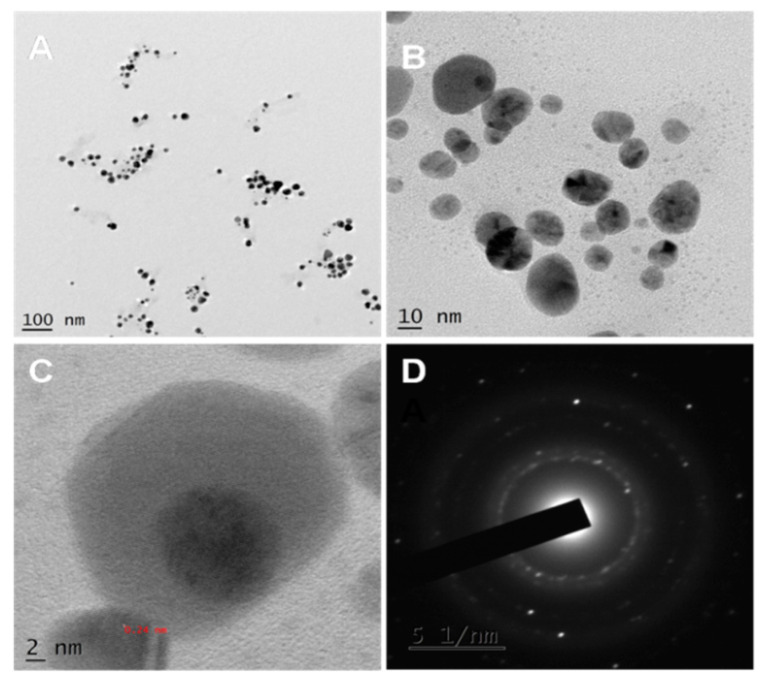
(**A**) TEM micrographs of green synthesized spherical AgNPs. (**B**) Green synthesized AgNPs in higher magnification (**C**) HRTEM image showing the interplanar distance of 0.24 nm (**D**) SAED pattern showing crystallinity of the AgNPs.

Figure 3A–D shows TEM images of green synthesized AgNPs, and it can be seen that most synthesized AgNPs are spherical. Triangular and quasi-spherical nanoparticles were also found. The nanoparticles synthesized using a biological method exhibited a wide range of shapes and sizes [34]. Since biomolecules cap the synthesized AgNPs, the edges of the nanoparticles are lighter than the core [35]. The particle size was estimated to be 10–30 nm. Figure 3C represents HRTEM (High-Resolution TEM) images with lattice fringes and an interplanar distance of 0.24 nm, attributed to (111) silver planes [36]. Figure 3D depicting the selected area electron diffraction pattern (SAED) of AgNPs shows concentric rings, suggesting that the green synthesized AgNPs are crystalline.

### 3.3. Antioxidant and Antimicrobial Activity of AgNPs

**Figure 4:**
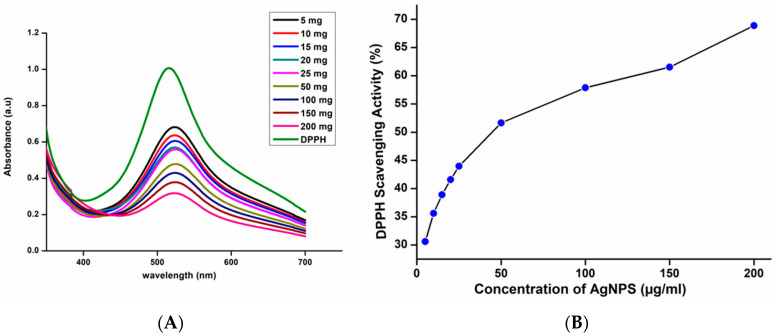
(**A**) UV–Visible spectra of DPPH with different concentrations of AgNPs (**B**) DPPH scavenging activity of AgNPs.

The stable free radical 1,1-diphenyl-2-picrylhydrazl (DPPH) has a purple color and a strong absorption peak at 517 nm. The free radical present in the DPPH is paired off in the presence of an antioxidant, lowering the absorbance and color intensity. AgNPs reduces DPPH radicals by giving them an electron or a proton [37]. Figure 4 depicts the DPPH scavenging activity of AgNPs. When the nanoparticle concentration was increased from 5 µg/mL to 200 µg/mL, the percentage of scavenging activity also increased gradually. Green synthesized AgNPs showed scavenging activity of 31% for the concentration of 5 µg/mL and 69% for the concentration of 200 µg/mL. Green synthesized AgNPs shows excellent antioxidant activity due to the presence of bioactive capping agents such as flavonoids and phenolic compounds on the surface of nanoparticles so that it can be used to prevent and cure degenerative diseases [38].

**Figure 5:**
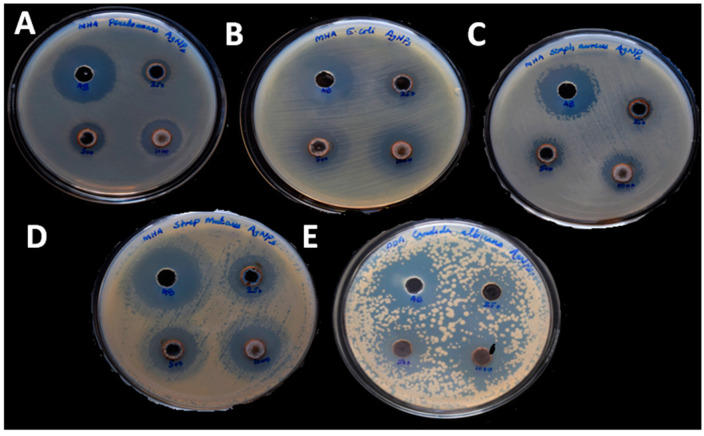
Antimicrobial activity of AgNPs against: (**A**) *Pseudomonas aeruginosa*(**B**) *Escherichiacoli* (**C**) *Staphylococcusaureus* (**D**) Streptococcus mutans (**E**) *Candida albicans*.

Green synthesized AgNPs have antibacterial and antifungal activity against human pathogenic microorganisms, i.e., *S. aureus*, *S. mutans*, *P. aeroginosa*, *E. coli*, and *C*. *albicans*, as shown in Figure 5. Among the four bacteria studied, *P. aeroginosa* and *S. mutans* have a maximum inhibition zone 20 mm, whereas *S. aureus* and *E. coli* have 19 mm for a concentration of 1000 µg/mL. Fungi *C*. *albicans* has shown azone of Inhibition of 16 mm for 1000 µg/mL concentration. We observed the increase of diameter of zone of inhibition with the increases of concentration of nanoparticles. AgNPs have effective antimicrobial properties due to their large surface area, which allows for an intimate proximity with microorganism cell walls [39]. It is toxic to a variety of pathogenic bacteria. It can be applied as an antimicrobial coating to various surfaces, including surgical equipment, biomimetic, wound dressing, fabrics, therapeutic and food containers, consumer goods, cleansing, purity monitoring appliances, and consumer products [40]. It was observed that the zone of inhibition created by AgNPs against both the Gram-negative and Gram-positive bacterial strains is more compared to the fungi C.albicans. This is primarily due to the variations in cell structure and organization. Eukaryotic yeast cells are more complex and have a superior detoxification system than prokaryotic bacterial cells to resist more concentrations of AgNPs [41]. The antifungal activity of AgNPs is attributed to the interaction of nanoparticles with the fungal cell wall and membrane, according to Kim et al. It causes membrane degradation and loss of integrity, as well as the formation of pores, which can lead to cell death [42].The Zone of inhibition of AgNPs against the human pathogen was given in Tables S1 and S2 (Supplementary Materials).

### 3.4. Characterization of Composite Films

**Figure 6:**
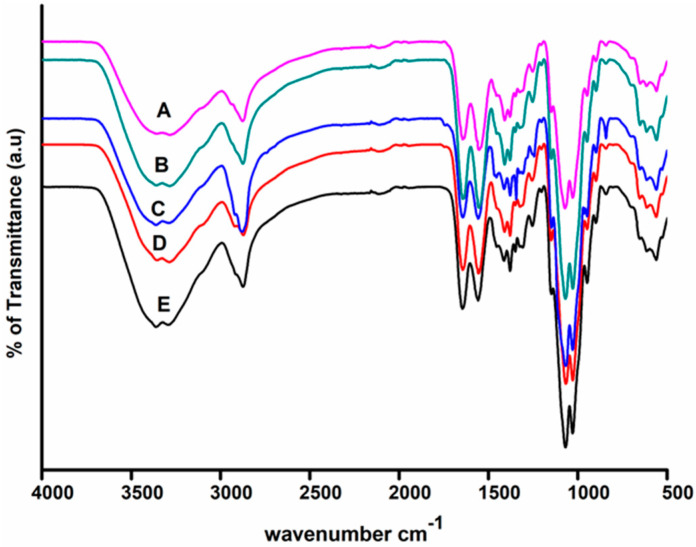
FTIR spectra of (**A**) CG (2% chitosan, 2% gelatin, 0.5% PEG, 0.075% AgNPs) (**B**) CG1 (2% chitosan, 2% gelatin, 0.5% PEG, 0.0075% AgNPs) (**C**) CG2 (2% chitosan, 2% gelatin, 0.5% PEG, 0.0125% AgNPs) (**D**) CG3 (2% chitosan, 2% gelatin, 0.5% PEG, 0.025% AgNPs) (**E**) CG4 (2% chitosan, 2% gelatin, 0.5% PEG, 0.05% AgNPs).

Figure 6 illustrates the FTIR spectra of nanocomposite films. The spectra of pure CG film show a broad band at 3320 cm^−1^, which can be attributed to the stretching vibrations of pendant groups such as NH_2_ and OH in the CH and GE, respectively [22]. The asymmetric stretching vibrations of CH_2_ groups of the CH chain are assigned to the IR band at 2874 cm^−1^ [43]. The IR band at 1640 cm^−1^ corresponding to C=O stretching, amide I, 1551 cm^−1^ attributed to N–H bending amide II [44], and 1345 cm^−1^ correspond to C–N stretching. The IR band at 1058 cm^−1^ is responsible for the C–O–C stretching of the polysaccharide chain’s linkage [22]. The chemical structure of CH did not affect by additives. Upon the inclusion of additives, there is no stipulate formation of a new bond; however, It has been observed that the peak’s intensity has increased [44]. It also indicates that the formation of coordination bonds between various entities of CH, GE, and AgNPs. The FTIR spectra of synthesized films agree well with previous findings [45].

**Figure 7:**
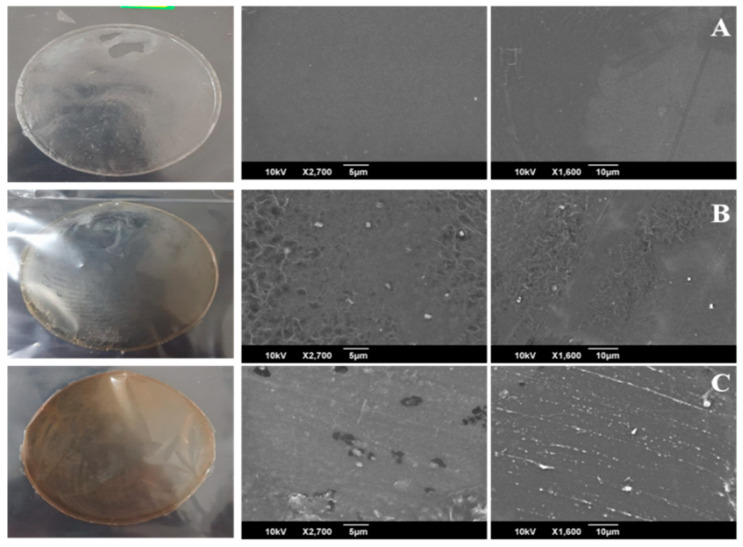
SEM images of (**A**) CG (**B**) CG1 (**C**) CG4.

The SEM micrographs of CG and CG4 composites films have represented in Figure 7. SEM images of plasticized CG films without nanoparticles are relatively smooth, good compatibility, and homogenous [23]. As we observed in Figure 7B,C, white spots on the polymer surface confirmed the existence of AgNPs on polymer matrix [22]. When the concentration of nanoparticles increases within the polymer matrix, we noticed the increase of white spots on the surface. When 0.05% of AgNPs added to the CH–GE matrix, the slight agglomeration of particles observed. The findings are similar to the previous report [22]. These nanoparticles were tightly adhered to the CH–GE matrix, potentially altering the physical and chemical properties of the films.

### 3.5. UV–Visible Spectroscopy and Opacity of the Films

**Figure 8:**
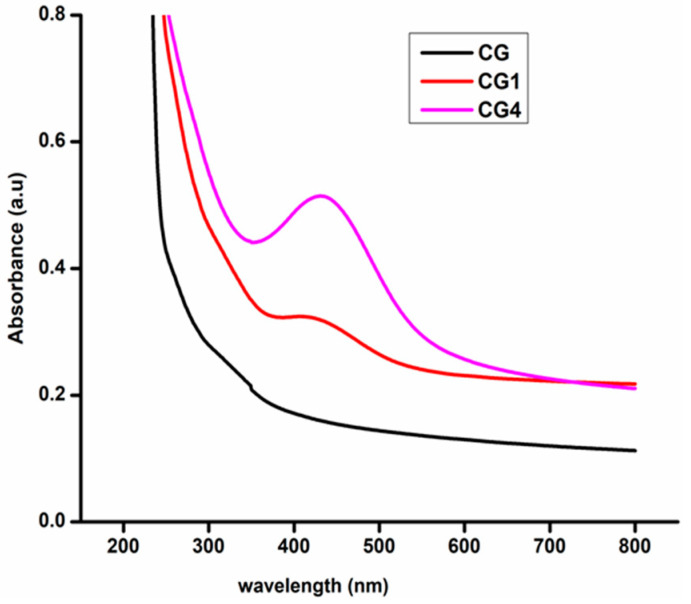
UV–Visible spectra of the synthesized nanocomposite films.

The UV–Vis absorption spectra of the films are depicted in Figure 8. The blank CG films didn’t show any absorbance in UV–Visible spectra, but with the addition of AgNPs, we noticed an absorbance peak between 390–470 nm [32]. When the concentration of AgNPs increases within the matrix, then the absorbance also gets increases. The opacity of the fabricated films was obtained from UV–Visible spectra by taking the absorbance at 600nm. Among the fabricated films, CG films show the least opaque and higher transparency than the other films. As the concentration of AgNPs in the CH–GE matrix increases, the films become more dark, opaque, and less transparent. A similar trend in the opacity values for the chitosan/ZnO/neem oil composite films was mentioned by Sanuja et al. [46]. The addition of nanoparticles prevents visible and ultraviolet light from penetrating the packaging film and perforating the food material within it, potentially preserving nutrients, color, and flavor [47]. Table 2 illustrates the opacity values of various fabricated films.

### 3.6. Film Thickness and Apparent Density

All fabricated films are homogenous and flexible, and it suggests good congruence among components of films, the thickness of the fabricated films ranged from 0.03–0.09 mm. The apparent density of films shows in the range of 0.11–0.25 g/cm^3^. Thickness and apparent density are represented in Table 2. The slight difference in apparent density can be due to the disparity in porosity and solid material density of films caused by different additives [23].

### 3.7. Evaluation of Mechanical Properties

The mechanical strength of composite films is a critical criterion for determining their ability to retain integrity against environmental circumstances along with packaging applications. Tensile strength (TS) and elongation at break (EAB) are given in Table 2. The TS of neat CH–GE film without the addition of AgNPs content is 24.47 ± 0.067 MPa, and the % of EAB is 4.48 ± 0.05. The incorporation of AgNPs slightly increased TS of the films and observed an opposite trend for % of EAB. With the addition of AgNPs, the tensile strength of the films increases; it may be due to the increases in crystallinity [29] or an intermolecular interaction between CH, GE, and AgNPs within the polymer matrix [30]. As the concentration of nanoparticles increases, we expect to raise the TS, but the films with 0.05 (wt.%) nanoparticles showed a decrease in TS. The surface of nanoparticles was stabilized by water-soluble phytochemicals present in the plant extract [16]. The number of hydrophilic groups increases as the concentration of AgNPs in the polymer matrix rises. In consequence, the flexibility of the films increases, and a decrease of tensile strength was observed. Mechanical strength of composite films are comparable with plastic films such as HDPE is 19–44 MPa, and LDPE is 22–23 MPa [22].

### 3.8. Swelling Degree and Solubility, Water Vapour Transmission Rate (WVTR), and Moisture Retention Capability (MRC)

**Figure 9:**
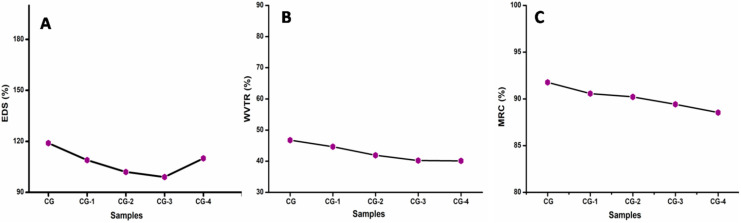
(**A**) Swelling Degree of films. (**B**) WVTR of films. (**C**) MRC of films.

The swelling behavior composite films were shown in Figure 9A. Incorporating the plasticizer PEG into the CH–GE matrix generates flexibility in the blended films and usually increases the swelling degree of films [44]. However, it was observed that adding AgNPs to the plasticized CH–GE matrix reduces swelling; this may be due to AgNPs binding to electron-abundant oxygen and nitrogen atoms of ether and amine groups of CH or GE, which offers an additional cross-link within the chain networks. The swelling of films is decreased due to the cross-linking action of AgNPs [48]. As the percentage of nanoparticles is increased to 0.05 (*w*/*v*%), there is a slight increase in swelling degree due to an oversaturation effect of the polymer network active points, which leads to a plasticizing effect in the matrix [49]. A higher crosslinker amount results in an increasing swelling ratio by increasing water molecule localization within the polymer network [50]. Solubility of the films (Table 2) slightly increases with the addition of AgNPs; this may occur due to the water-soluble phytochemicals such as carbohydrates, alkaloids, tannins, polyphenols [21] present on the surface of nanoparticles.

The rate at which water vapor passes through the film is known as the water vapor transmission rate. For applications that need effective polymer barriers, such as food packaging, protective coatings and so on, determining the WVTR of nanocomposite films is essential. The WVTR of plasticizer added films is consistently higher because it reduces the intermolecular interaction between the polymer chains and increases the molecular spaces between the polymer matrixes, leading to enhanced permeability through plasticized films [51]. AgNPs have a cross-linking effect in the CH–GE matrix, causing polymeric chains to become more compact, reducing interstitial spaces in the polymer matrix, and slowing water molecule diffusion through the films [23]. As a result, the WVTR of the films decreases. Incorporation of AgNPs to the polymer matrix causes reduction of WVTR described in Rhim et al. [52]; the WVTR of montmorillonite-loaded cellulose acetate/polyethyleneglycol film was also reduced when the nanoparticle concentration was increased, according to Saha et al. [53]. Food packaging materials should have a low WVTR to prevent moisture exchange among the food and the nearby environment [14]. The Water Vapor Transmission Rate (WVTR) of various films is shown in Figure 9B.

The loss of water vapors from the films is determined by moisture retention capacity. MRC of the fabricated films shows the range of 88–92%; results indicate adequate MRC of nanocomposite films (Figure 9C). All the nanocomposites films show almost similar MRC values regardless of their compositions [54]. Since the presence of the plasticizer PEG in the fabricated films creates more intermolecular spaces between polymeric chains, there are still more spaces available in the matrix for absorbing moisture [22]. With the addition of AgNPs, there was a slight decrease in moisture retention capability due to the cross-linking action of AgNPs within the CH–GE matrix [23]. The values are based mostly on moisture levels that must be preserved for foods and fruits between 78% and 95% [29].

Mean, Standard Deviation, standard Error of Swelling Degree, Water Vapor Transmission Rate, Moisture Retention Capability were given Tables S8–S10 (Supplementary Materials), respectively.

### 3.9. Biodegradability of CH–GE Nanocomposites Films

**Figure 10:**
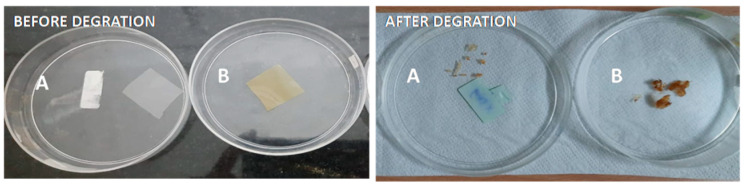
Biodegradation studies of films: (**A**) CG (**B**) CG4.

**Figure 11:**
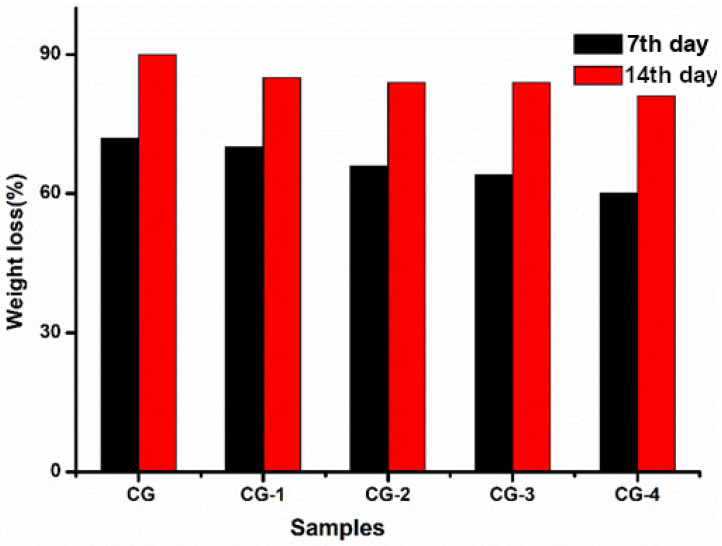
Soil biodegradability nanocomposite films over a period of time.

The degradation of the composite films in soil was studied to evaluate their biodegradability in natural conditions. It was observed that, when the CG composite films were exposed to the soil for 14 days, the films become fragile and hard and shrink (Figure 10). Figure 11 depicts the film’s biodegradation rates over time as determined by weight loss. The soil’s moisture could easily seep into the polymer network and weaken the polymer chains; hence, it is easily attacked by soil microorganisms [30]. After 14 days, CG composite films showed the highest percentage of degradation; as the percentage of AgNPs increases in the matrix, the degradation of films decreases may be due to the antimicrobial activity of AgNPs [55].

### 3.10. Potential Applications of the Synthesized Film and Its Antimicrobial Ability

**Figure 12:**
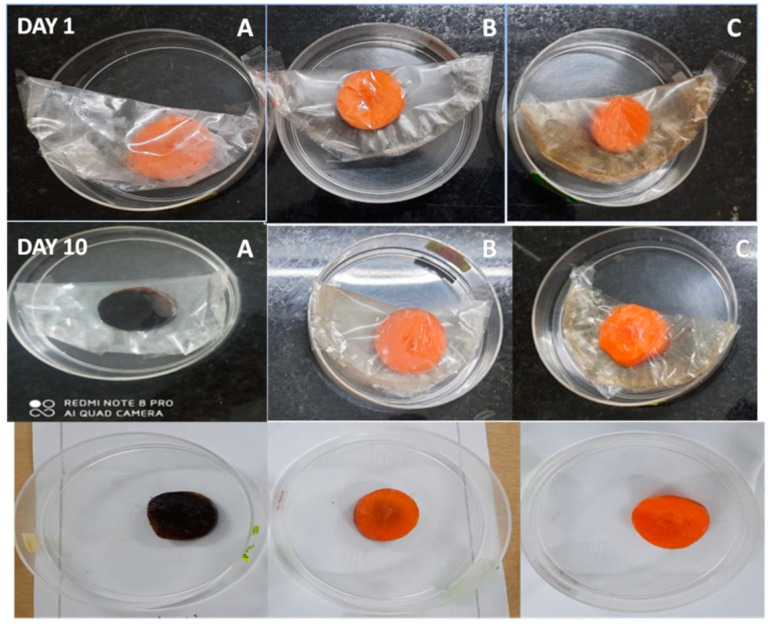
Visual appearances of carrot samples during storage: (**A**) Polyethylene films (**B**) CG (**C**) CG4.

**Figure 13:**
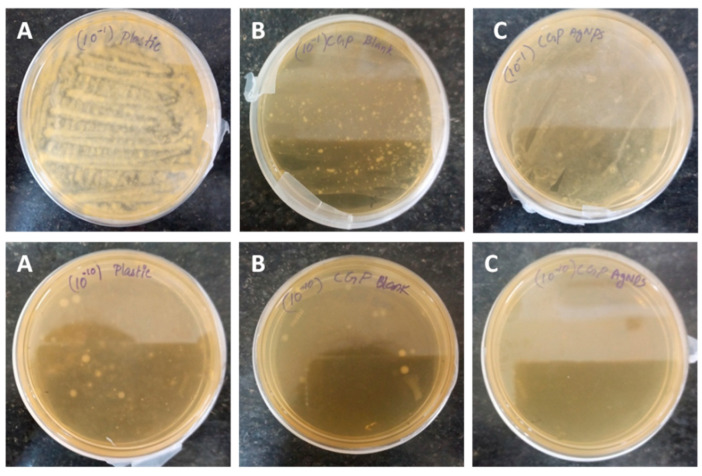
The antibacterial ability of composite films was evaluated through the spread plate method after 10 days of storage: (**A**) Polyethylene films (**B**) CG (**C**) CG4.

To check the real-time applications of the composite film, bacterial resistance studies were performed on carrot pieces. Carrot pieces were wrapped in commercial polyethylene (PE), CG, and CG4 films; observed visual changes of carrot pieces over a ten days of storage and are represented in Figure 12. The carrot piece covered by polyethylene films has become blackish after ten days of storage due to the microbial action, but these changes are not observed in the carrot piece were wrapped by CG and CG4 films. It may be due to the antimicrobial activity of both chitosan [56] and AgNPs [57]. The carrot packed with CG and CG4 films seemed fresh, with no signs of foulness or mildew. Antibacterial activity (Figure 13) was analyzed using the spread plate method, and the values were measured in terms of CFU values. The carrot pieces wrapped with CG4 films were colonized to the least extent, obtaining CFU values of 10 × 10^10^ for the dilution 10^−10^, but the CFU values of CG films and polyethylene films were 14 × 10^10^ and 80 × 10^10^, respectively, for the same dilution. This result suggested the magnificent antimicrobial efficiency of CG4 films compared to that of commercial films. CFU values all the films were given Table S3 (Supplementary Materials). These findings indicate that the prepared CH–GE–AgNPs hybrid films have the potential to act as a novel food packaging material with anti-microbial activity, which helps to safeguard the food for long time.

## 4. Conclusions

We successfully produced packaging films using naturally derived biodegradable polymer materials CH and GE to reduce the environmental harm caused by synthetic plastic packaging materials. Antioxidant and antibacterial AgNPs were used as reinforcing filler in the CH–GE matrix. The synthesized nanocomposite packaging films were characterized using UV–Visible spectroscopy, FTIR, and SEM. The presence of AgNPs within the CH–GE matrix is confirmed using UV–Visible studies; it also describes the opacity of the composite films. CH–GE films were the least opaque, and the opacity of films increased upon the addition of AgNPs. FTIR studies of composite films revealed the possible interactions between the AgNPs and matrix; the result also reveals no chemical bond formed upon the inclusion of additives. The SEM images clearly show the homogeneity of different components in the matrix. Mechanical properties of films slightly increase upon the addition AgNPs. It may be due to the cross-linking action of AgNPs, which in turn increases the compactness of the polymer matrix and leads to a decrease in MRC, Swelling degree, and WVTR. All the films were biodegradable, and we noticed the changes over 14 days; the size of the film was diminished in soil, indicating spontaneous biodegradation in the soil environment. AgNPs added films showed the least biodegradation due to their antimicrobial activity. CH–GE–AgNPs films emerged as a promising antimicrobial packaging material that can expand the shelf life of carrot pieces.

Chitosan and gelatin based packaging materials lengthen the shelf life of vegetables, fruits, and other farm commodities while also reducing synthetic plastics and promoting healthy foods and a pleasant environment. Future studies might focus on large-scale manufacture and implementation of biopolymer-based food packaging materials to lessen the impact of artificial plastics on the environment. Different substances such as crosslinker, strengthening agents, and a few distinctive components with antimicrobial, antioxidant, and much less cytotoxicity improve the functional properties of a biopolymer and based films device can be used for different applications such as edible films, coatings and so forth.

## Figures and Tables

**Table 1:** Composition of polymer nanocomposite films.

Sample	CH (*w*/*v*%)	GE (*w*/*v*%)	PEG (*w*/*v*%)	AgNPs (*w/v*%)
CG	2	2	0.5	0.0
CG1	2	2	0.5	0.0075
CG2	2	2	0.5	0.0125
CG3	2	2	0.5	0.025
CG4	2	2	0.5	0.05

**Table 2:** Opacity, Film Thickness, Apparent Density, Tensile Strength (TS), Percentage of Elongation at Break (EAB %), Solubility. Mean values and standard deviation are given.

Films	Opacity (mm^−1^)	Film Thickness (mm)	Apparent Density (gm/cm^3^)	TS (MPa)	EAB (%)	Solubility (%)
CG	1.47 ± 0.05	0.03± 0.005	0.11± 0.015	24.47 ± 0.067	4.48 ± 0.05	42.92 ± 0.64
CG1	1.60 ± 0.08	0.05 ± 0.005	0.17 ± 0.015	25.80 ± 0.1	4.34 ± 0.05	44.98 ± 0.19
CG2	2.25 ± 0.08	0.05 ± 0.0	0.16 ± 0.005	26.30 ± 0.25	4.29 ± 0.04	48.97 ± 0.65
CG3	3.49 ± 0.08	0.08 ± 0.011	0.16 ± 0.005	26.40 ± 0.05	4.12 ± 0.05	51.54 ± 0.54
CG4	4.93 ± 0.06	0.09 ± 0.0	0.25 ± 0.02	24.39 ± 0.05	4.50 ± 0.06	52.60 ± 0.50

## Data Availability

The data presented in this study are available on request from the corresponding author.

## Supplementary Materials

The following are available online at https://www.mdpi.com/article/10.3390/polym13111680/s1, Figure S1: Green synthesized nanoparticles colour change observed from pale yellow to dark yellow then finally to reddish brown, Figure S2: X-ray diffraction patterns of AgNPs synthesized using M.frondosa leaf extract, Table S1: Anbacterial activity of AgNPs, Table S2: Antifungal activity of AgNPs (*Candida albicans*), Table S3: Colony forming units of bacterial suspension, Table S4: Thickness of the films, Table S5: Apparent density of the films, Table S6: Opacity of the films, Table S7: Tensile strength of the films, Table S8: Elongation at break, Table S8: Swelling degree of films, Table S9: WVTR of films, Table S10: Moisture retention capability of films, Table S11: Solubility of films, Equation S1: Opacity of the films, Equation S2: Colony-forming unit.
