# Supplementary material for: Chitosan/Gelatin/Silver Nanoparticles Composites Films for Biodegradable Food Packaging Applications
Source: Polymers (Basel). 2021 May 21;13(11):1680. doi: 10.3390/polym13111680 (PMC8196760; doi:10.3390/polym13111680)
Supplement: Supplementary file 1 [file polymers-13-01680-s001.zip › polymers-1218942-supplementary.pdf]

polymers

MDPI

Supplementary Materials

Chitosan/Gelatin/Silver Nanoparticles Composites Films for Biodegradable Food Packaging Applications

Sreelekha *Ediyilyam* [1], **Bini George** [1,*], **Sarojini Sharath Shankar** [2,5,*], Dennis Thomas T [3], **Stanisław Wacławek** [4], **Miroslav Černík** [4,*] **and Vinod V.T. Padil** [4,*]

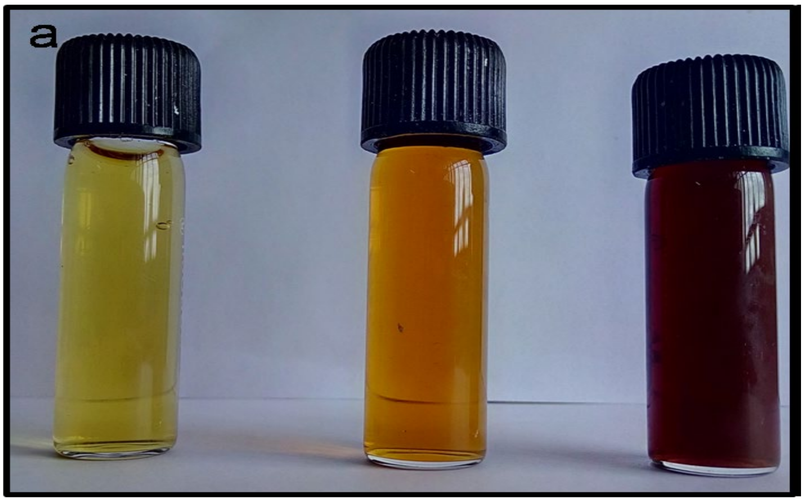

Figure S1. Green synthesized nanoparticles colour change observed from pale yellow to dark yellow then finally to reddish brown.

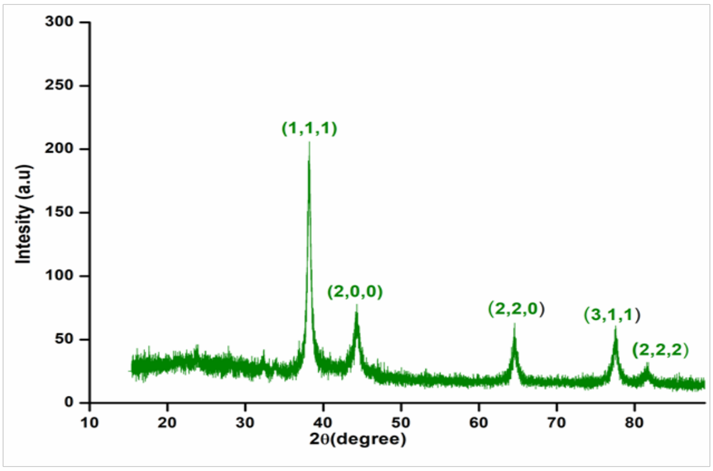

Figure S2. X-ray diffraction patterns of AgNPs synthesized using M.frondosa leaf extract. The diffraction data acquired were in agreement with the literature reports of FCC structure of silver (JCPDS file no. 04–0783).

Table S1. Anbacterial activity of AgNPs.

Sample concentration (µg/ml)	Zone of inhibition (mm)			
	S. Aureus		*S. Mutans*	
Streptomycin (100 µg)	26	28	27	28
250	12	14	15	14
500	15	17	17	17
1000	19	20	19	20

Table S2. Antifungal activity of AgNPs (Candida albicans).

Sample concentration (µg/ml)	Zone of inhibition (mm)
Clortimazole (100 µg)	26
250	11
500	13
1000	16

Table S3. Colony forming units of bacterial suspension from carrots stored in different films.

Carrot study in different films	Colony forming units (CFU/mL)
Plastic films	$80x10^{10}$
CG	$14x10^{10}$
CG4	$10x10^{10}$

Equation S1: Opacity of the films is calculated by taking the absorbance at 600nm using UV-Visible spectroscopy. Then, the opacity was calculated using the following equation:

$$O = \frac{\mathbf{Abs600}}{L}$$

Where, O is the opacity, Abs600 is the absorbance value at 600 nm and L is the film thickness (mm).

Equation S2 colony-forming unit (**CFU** or **cfu**) is a measure of viable bacterial or fungal cells CFU/ml can be calculated using the formula:

$$cfu/ml = (No.\ of\ colonies\ x\ dilution\ factor)/volume\ of\ culture\ plate$$

Table S4. Thickness of the films.

Films	Thickness of the films					
	x	y	z	Average	SD	SE
CG	0.03	0.03	0.04	0.033	0.005	0.002
CG1	0.05	0.05	0.04	0.046	0.005	0.002
CG2	0.05	0.05	0.05	0.05	0.00	0.00
CG3	0.08	0.06	0.08	0.073	0.011	0.005
CG4	0.09	0.09	0.09	0.09	0.00	0.0

SD- standard deviation, SE- Standard error

Table S5. Apparent density of the films.

Films	Apparent density of the films					
	x	y	z	Average	SD	SE
CG	0.11	0.13	0.1	0.1133	0.015	0.008
CG1	0.17	0.15	0.18	0.1666	0.015	0.008
CG2	0.16	0.16	0.16	0.16	0	0
CG3	0.16	0.15	0.16	0.156	0.0057	0.003
CG4	0.25	0.23	0.27	0.25	0.02	0.011

Table S6. Opacity of the films.

Films	Opacity of the films					
	x	y	z	Average	SD	SE
CG	1.47	1.4	1.5	1.456	0.051	0.029
CG1	1.6	1.51	1.68	1.596	0.085	0.049
CG2	2.25	2.4	2.32	2.323	0.075	0.043
CG3	3.49	3.56	3.41	3.486	0.075	0.043
CG4	4.94	4.88	4.99	4.936	0.055	0.031

Table S6. Tensile strength of the films.

Films	Tensile strength of the films					
	x	y	z	Average	SD	SE
CG	24.4	24.4	24.5	24.4	0.066	0.03
CG1	25.8	25.9	25.7	25.8	0.1	0.05
CG2	26.3	26.6	26.1	26.3	0.25	0.14
CG3	26.4	26.4	26.5	26.4	0.05	0.03
CG4	24.4	24.4	24.5	24.4	0.06	0.03

Table S7. Elongation at break.

Films	EAB (%) of the films					
	x	y	z	Average	SD	SE
CG	4.4	4.5	4.4	4.4	0.05	0.02
CG1	4.3	4.2	4.3	4.3	0.05	0.02
CG2	4.2	4.2	4.3	4.2	0.04	0.02
CG3	4.1	4.0	4.1	4.1	0.05	0.02
CG4	4.5	4.4	4.5	4.5	0.06	0.03

Table S8. Swelling degree of films.

Films	Swelling degree of the films					
	x	y	z	Average	SD	SE
CG	119	119	120	119.3	0.57	0.33
CG1	109	108	109.9	108.9	0.95	0.54
CG2	102	102	101	101.6	0.57	0.33
CG3	99	99	98	98.6	0.57	0.33
CG4	110	109	110	109.6	0.57	0.33

Table S8. WVTR of films.

Films	WVTR of the films					
	x	y	z	Average	SD	SE
CG	46.7	46.7	46.7	46.7	0.045	0.026
CG1	44.6	44.7	44.6	44.6	0.03	0.020
CG2	41.9	41.9	41.8	41.8	0.015	0.008
CG3	40.2	40.2	40.2	40.2	0.005	0.003

CG4	40.1	40.1	40.1	40.1	0.026	0.015

Table S9. Moisture retention capability of films.

Films	MRC of the films					
	x	y	z	Average	SD	SE
CG	91.7	91.7	91.7	91.7	0.020	0.012
CG1	90.5	90.5	90.6	90.56	0.04	0.023
CG2	90.2	90.2	90.2	90.2	0.005	0.003
CG3	89.4	89.4	89.4	89.4	0.011	0.006
CG4	88.5	88.5	88.5	88.5	0.015	0.008

Table S10. Solubility of films.

Films	Solubility of films					
	x	y	z	Average	SD	SE
CG	42.9	43.5	42.2	42.9	0.64	0.36
CG1	44.9	45.1	44.7	44.9	0.19	0.11
CG2	48.9	49.6	48.3	48.9	0.65	0.37
CG3	51.5	52.0	51.0	51.5	0.54	0.31
CG4	52.6	52.8	51.9	52.4	0.50	0.28
